# The distribution of some extremum on the risk process whose income depend on the current reserve

**DOI:** 10.1186/s40064-016-3664-5

**Published:** 2016-11-15

**Authors:** Jingmin He, Zaiming Liu, Wei Zhang

**Affiliations:** 1 School of Science, Tianjin University of Technology, Tianjin, 300384 China; 2 School of Mathematics and Statistics, Central South University, Changsha, 410083 Hunan China

**Keywords:** Ruin probability, Strong Markov property, Supreme profit and deficit, The time of ruin, 60J25, 91B30

## Abstract

This paper considers the distribution of some extremum on the risk process whose income depend on the current reserve. We first construct the defective renewal sequence and obtain the density function of them. By the presented renewal measure and the strong Markov property, the distribution of the first hitting time is obtained explicitly. Then, the ruin probability and the probability that the surplus process less than *x* is obtained. Furthermore, the distribution of supreme profits before ruin, the joint distributions of the supreme profit and the deficit before the time of the surplus process first up-crossing level zero after ruin, and the joint distributions of the supreme profit and the deficit before the surplus process leave zero ultimately are derived. Finally, the exact calculating results for them are obtained when the individual claim amounts in the compound Poisson risk model are exponentially distributed.

## Background

Before introducing the model, we revisit some important risk models. Assume that the claim arrival process $\{N(t)\}_{t\ge 0}$ is a Poisson process with parameter $\lambda$, and that the claim sizes $\{Z_{k}\}_{k\ge 1}$ independent of $\{N(t)\}_{t\ge 0}$, are positive, independent and identically distributed random variables with common density function *f* and mean value $\mu$. Then the well-known classical compound Poisson risk model is given by

$$\begin{aligned} U(t)=u+ct -\sum \limits _{i=1}^{N(t)}Z_{i}, \end{aligned}$$

where *u* denotes the initial capital of an insurance company, $c>0$ is the premium income rate. If the company charges a constant premium rate *c*, but invests its money at interest rate $\delta$, we get the compound Poisson risk model with constant interest. The dynamics of the surplus process can be described by

$$\begin{aligned} U(t)=u+\int _{0}^{t}(c+\delta U(s))ds-\sum \limits _{i=1}^{N(t)}Z_{i}. \end{aligned}$$

The risk models above have a common characteristic, that is, the surplus process moves according to the same differential equation in between jumps. All these models and many other risk models modified from the compound Poisson risk model belong to the risk process whose income depend on the current reserve (other terms are level-dependent risk processes, see Chapter 8 of Asmussen and Albrecher 2010). The evolution of the surplus process can be expressed as

1 $$\begin{aligned} U(t)=u+\int _{0}^{t}g(U(s))ds -\sum \limits _{i=1}^{N(t)}Z_{i}, \end{aligned}$$

where $g: R\longmapsto R$ is a continuously differentiable Lipschitz function which represents the reserve-dependent income rate. Denoted by $\{S_{n}\}_{n\ge 1}$, the sequence of the claim times and $\{T_{k}\}_{k\ge 1}$, the inter-arrival i.e. the time periods between successive claims. Then we have

$$\begin{aligned} S_{0}=0,\quad T_{k}=S_{k}-S_{k-1}. \end{aligned}$$

The stochastic nature of the model (1) is mainly derived from the compound Poisson process. Between successive claim arrival epochs, the process follows a deterministic path, and satisfies the same differential equation, while the uncertainty of the claim determines the uncertainty of the initial value of the differential equation. Starting from the initial value *u*, the risk process run in accordance with a differential equation. When the claim occurs, the risk process run from the new initial value, according to the same differential equation, and the procedure goes on and on. We use $\phi (t,x)$ to denote the deterministic path starting from the initial value *x* in between jumps, which satisfies

$$\begin{aligned} \left\{ \begin{array}{ll} \displaystyle {\frac{d\phi (t,x)}{dt}}=g(\phi (t,x)),\quad t>0.\\ \phi (0,x)=x.\\ \end{array}\right. \end{aligned}$$

In addition, we assume that there exists a constant $\varepsilon >0$ such that $\inf \nolimits _{x\in R} g(x)\ge \lambda \mu +\varepsilon$, which ensures that the ruin probability is less than 1 and $P^{u}(\lim \nolimits _{t\rightarrow \infty }U(t)=\infty )=1$. In this paper we use $P^x(\cdot )$ to denote $P(\cdot |x\in \mathbb {R})$ the probability of the process $\{U(t)\}_{t\ge 0}$ with initial value *x* generated on $(\Omega ,{\mathscr {F}}_{\infty })$.

For the reason why the model (1) is important, the readers are referred to Cai et al. (2009) and Chapter 8 of Asmussen and Albrecher (2010), where some well-known important risk models are given by taking different kinds of functions of $g(\cdot )$.

In fact the above model was considered by many authors: such as Asmussen (2000), Asmussen and Albrecher (2010), Cai et al. (2009), Dassios and Embrechts (1989), Das and Mahavier (2012), Embrechts and Schmidli (1994), Egídio dos Reis (2002), Li and Lu (2013) and Wang et al. (2003). For this model, Cai et al. (2009) investigate various applications of the total discounted operating costs up to default. Dassios and Embrechts (1989) or Embrechts and Schmidli (1994) showed in general how to use the theory of piecewise deterministic Markov processes for solving insurance risk problems. Das and Mahavier (2012) study the joint distribution of the surplus immediately before ruin and the deficit at ruin for the compound Poisson risk model with constant interest. Li and Lu (2013) consider the generalized expected discounted penalty function in a risk process with credit and debit interests. It is worthwhile pointing out that a deep review and details on applications of this model can be found at Chapter 8 of Asmussen and Albrecher (2010). From Dassios and Embrechts (1989) or Embrechts and Schmidli (1994), we know that $\{U(t)\}_{t\ge 0}$ is a piecewise deterministic Markov process. We use $P(t,x,\Gamma )$ to denote the transition function of the model (1), for any $\Gamma \in {\mathscr {B}}(\mathbb {R})$ (the Borel $\sigma$-algebra on $\mathbb {R}$). Throughout this paper, it is assumed that $P(t,x,\Gamma )$ has a density function *p*(*t*, *x*, *y*) for $y<\phi (t,x)$.

To the best of our knowledge, there are only a few papers exclusively concerned with the extremum of the risk model (1). The distribution of extremum is very important in risk theory, which can portray the best and worst condition of an insurance company and provide early warning for the development of the insurance company. It is worth pointing out that our method is different from the traditional method, and we obtain the distribution of the first hitting time by the method of constructing the renewal sequence. And then, the distributions of some extreme value are investigated.

Inspired by Wu et al. (2003), we mainly study the first hitting time and some extremum of the model (1). The rest of this paper is organized as follows. We intend to introduce in “Preliminaries” section the renewal measure of the defective renewal sequence. In “The first hitting time” section, the expression of the renewal measure is derived. Thus, the distribution function on the first hitting time is obtained. Furthermore, the ruin probability and the probability that the surplus process is less than *x* is obtained. In “The supreme profit and the deficit” section, the distribution of supreme profits before ruin, the joint distributions of the supreme profit and deficit before the time of the first up-crossing level zero after ruin, and the joint distributions of the supreme profit and deficit before the time of the surplus process leaving zero ultimately are derived. As a validation of all results’ applications, we give the explicit expressions for the compound Poisson risk model with the claim amount being exponentially distributed.

## Preliminaries

In this section, we first give some notation and terminologies, and then introduce the renewal measure. Let *T* be the time of ruin, $T_{x}$ the time of the surplus process below the level *x*, i.e. the time of default (see Cai et al. 2009) for the first time and *L* the time of the surplus process leaving zero ultimately, then

$$\begin{aligned}&T=\inf \{t\ge 0:U(t)<0\},\quad (T=\infty \quad {\text{if the set is empty}}),\\&T_{x}=\inf \{t\ge 0:U(t)<x\},\quad (T_{x}=\infty \quad {\text{ if the set is empty}}),\\&L=\sup \{t\ge 0:U(t)<0\}=\sup \{t\ge 0:U(t)=0\},\quad (L=0 \quad {\text{if the set is empty}}). \end{aligned}$$

From $P^{u}(\lim \nolimits _{t\rightarrow \infty }U(t)=\infty )=1$, we see that $P(L<\infty )=1$ and that the surplus will never become negative after time *L*.

Define the sequence of the *x* points on the time scale of the surplus process as follows:

$$\begin{aligned} T_1^{x}=\inf \{t>0: U(t)=x\}, \quad (T_1^{x}=\infty \hbox { if the set is empty).} \end{aligned}$$

**Fig. 1:**
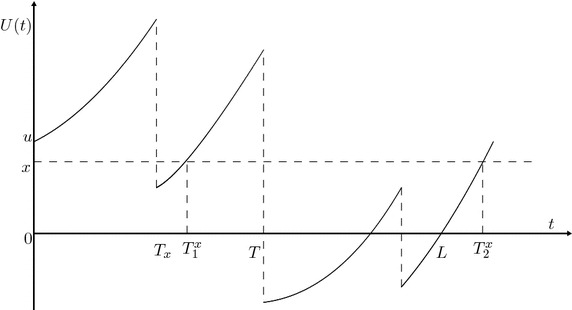
A typical realization of the risk process

In general, for $k=2,3,\ldots$, we recursively define

$$\begin{aligned} T_k^{x}=\inf \left\{t>T_{k-1}^{x}:U(t)=x\right\}, \quad (T_k^{x}=\infty \hbox { if the set is empty),} \end{aligned}$$

as shown in Fig. 1. For convenience, let $T_{0}^{x}=0$.

For every $t>0$, set

$$\begin{aligned} N_t^{x}=\sup\left \{k>0,T_k^{x}\le t\right\},\quad (N_t^{x}=0 \hbox { if the set is empty).} \end{aligned}$$

We see that $N_t^{x}$ is the number of *x* points before *t* (and including *t*). Therefore, $\{N_t^{x}\}_{t\ge 0}$ is a counting process and $N_\infty ^{x}=\sup \{k>0: T_k^{x}<+\infty \}\quad (N_\infty ^{x}=0$ if the set is empty) is the total number of the *x* points of the process. Putting ${\mathscr {F}}_t=\sigma \{U(s),s\le t\}$, then *T*, $T_{x}$ and $\{T_k^{x}\}_{k\ge 1}$ are all ${\mathscr {F}}_t$-stopping times.

It is known that (see, for example, Gerber and Shiu 1998) the stopping times play an important role in many risk portfolio. Among others, we only mention a few, for example Dickson and Li (2013), Gerber (1990), Gerber and Shiu (1998), Zacks (2007), Xu (2012), Kyprianou (2013), Landriault and Shi (2014), Li and Lu (2014), Li et al. (2015) and references therein.

For $$U(0)=u\ge 0$$, let

$$\begin{aligned} \Psi (u)=P^u(T<\infty ) \end{aligned}$$

be the probability of ruin with initial capital *u*, and $\Phi (u)=1-\Psi (u)$ be the survival probability. We define the probability the surplus process falls below the level *x*, i.e. the probability of default, as

$$\begin{aligned} \Psi _{x}(u)=P^u(T_{x}<\infty ), \end{aligned}$$

and the probability that the surplus process never falls below the level *x* can be expressed as

$$\begin{aligned} \Phi _{x}(u)=1-\Psi _{x}(u). \end{aligned}$$

For $k\ge 1$, we define

$$\begin{aligned} S_k^{x}=\left\{ \begin{array}{ll} T_k^{x}-T_{k-1}^{x},&\quad T_{k-1}^{x}<\infty , \\ \infty , &\quad {\mathrm {otherwise}}. \\ \end{array} \right. \end{aligned}$$

Since the process $\{U(t)\}_ {t\ge 0}$ has strong Markov property, we can verify that $\{S_{k}^{x}\}_{k\ge 1}$ are independent and that $\{S_{k}^{x}\}_{k\ge 2}$ is a sequence of $\mathrm {i.i.d.}$ random variables. Therefore, $\{N_t^{x}\}_{t\ge 0}$ is a renewal process. The *k*-th renewal epoch is $T_k^{x}=\sum \nolimits _{n=1}^{k}S_n^{x}$. Let $F_{x}$ be the common distribution of $\{S_{k}^{x}\}_{k\ge 2}$, and $F_{u}^{x}$ be the distribution of $S_{1}^{x}$. Then the renewal measure $G_{u}^{x}$ is defined by

2 $$\begin{aligned} G_{u}^{x}(t)=\sum \limits _{k=1}^{\infty }P^u(T_k^{x}\le t)=\sum \limits _{k=1}^{\infty }F_{u}^{x}*F_{x}^{(k-1)*}(t), \end{aligned}$$

where $*$ denotes the convolution and $F_{x}^{n*}(t)$ denotes the *n*-fold convolution of $F_{x}(t)$. Thus we have

$$\begin{aligned} G_{u}^{x}(I)=\sum \limits _{k=0}^{\infty }F_{u}^{x}*F_{x}^{k*}(I),\quad I\subset [0,\infty ), \end{aligned}$$

where *I* denotes a general interval. Let $g_{u}^{x}(\cdot )$ and $f_{u}^{x}(\cdot )$ be the density functions of $G_{u}^{x}$ and $F_{u}^{x}$ respectively, if they exist.

## The first hitting time

We now show in detail how the renewal measure $G_{u}^{x}(\cdot )$ can be used to express the first hitting time. The key point is to obtain $G_{u}^{x}(\cdot )$. We first give the following lemma, which plays an important role in getting the expression of $G_{u}^{x}(\cdot )$.

### **Lemma 1**

*Let* $X_{t}$ *satisfy the ordinary differential equation*

$$\begin{aligned} \frac{dX_{t}}{dt}=h(X_{t}),\quad {\text{for any}}\ t\in [0,T], \end{aligned}$$

*where* $h(\cdot )$ *is a continuously differentiable Lipschitz continuous function. If there exists a* $t^{*}\in [0,T]$, *such that* $X_{t^{*}}=x$, *then we have*

$$\begin{aligned}&|X_{s}-X_{0}|\le K_{1}\cdot s,\quad {\text{for any}}\ s\in [0,T].\\&|X_{T}-X_{0}-h(x)T|\le KK_{1} T^{2}, \end{aligned}$$

*where constant* $K_{1}$ *depends only on* *T* *and* *x* *and constant* *K* *depends on* $h(\cdot )$.

### *Proof*

Without loss of generality we assume that $h(x)>0$. Note that $X_{t^{*}}=x$, then $X_{s}$ is bounded in $s\in [0,T]$. Since $h(\cdot )$ is a continuous function, then there exists a constant $K_{1}$ depending only on *T* and *x*, such that for any $0\le s\le T$, $|h(X_{s})|\le K_{1}$. Hence, for any $s\in [0,T]$, we obtain

$$\begin{aligned} |X_{s}-X_{0}|=\Big |\int _{0}^{s}h(X_{l})dl\Big |\le \int _{0}^{s}|h(X_{l})|dl \le K_{1}\cdot s. \end{aligned}$$

Thus we have

$$\begin{aligned} |X_{T}-X_{0}-h(x)T|&= |X_{T}-X_{0}-h(X_{t^{*}})T|=\Big |\int _{0}^{T}h(X_{l})-h(X_{t^{*}})dl\Big |\\ &\le {} \int _{0}^{T}|h(X_{l})-h(X_{t^{*}})|dl\le \int _{0}^{T}K|X_{l}-X_{t^{*}}|dl\\&\le {} K\int _{0}^{T}K_{1}|l-t^{*}|dl \le KK_{1} T^{2}. \end{aligned}$$

This ends the proof. $\square$

By the description just after the model (1), we know that the function $g(\cdot )$ of model (1) can be considered as special cases of the function $h(\cdot )$ of Lemma 1.

### **Theorem 1**

*For* $s\ge 0$, *we have*

1. *When* $$\phi (s,u)>x$$,
  3 $$\begin{aligned} g_{u}^{x}(s)=\left\{ \begin{array}{ll} g(x)p(s,u,x) &{}\quad {\mathrm{if}}\ s>0,\\ 0 &{}\quad \mathrm{{if}}\ s=0,\\ \end{array}\right. \end{aligned}$$
  *where* $g(\cdot )$ *is given by* (1).
2. *When* $$\phi (s,u)<x$$,
  $$\begin{aligned} g_{u}^{x}(s)=0. \end{aligned}$$
3. *When* $$\phi (s,u)=x$$,
  - *If* $s>0$, *then* $G_{u}^{x}(t)$ *will jump at time* *s*, *that is*,
    $$\begin{aligned} G_{u}^{x}(t)=0,\quad \mathrm{{for}}\ 0\le t<s,\quad G_{u}^{x}(s)=e^{-\lambda s}. \end{aligned}$$
  - *If* $$s=0$$, *i.e.*, $$s=0, u=x$$, *then*
    $$\begin{aligned} g_{u}^{x}(s)=0. \end{aligned}$$

### *Proof*

1. When $\phi (s,u)>x$, we have
  $$\begin{aligned} g_{u}^{x}(s)ds&= {} \sum \limits _{k=1}^{\infty }P^{u}\left( T_{k}^{x}\in (s,s+ds]\right) =\sum \limits _{k=1}^{\infty }P^{u}\left( T_{k}^{x}\in ds\right) \\&= {} \sum \limits _{k=1}^{\infty }P^{u}\left( T_{k}^{x}\in ds, N(s,s+ds]=0\right) +\sum \limits _{k=1}^{\infty }P^{u}\left( T_{k}^{x}\in ds, N(s,s+ds]\ge 1\right) . \end{aligned}$$
  By its probability meaning, we obtain
  $$\begin{aligned} \sum \limits _{k=1}^{\infty }P^{u}\left( T_{k}^{x}\in ds, N(s,s+ds]=0\right) =P^{u}\left( U(s)<x, U(s+ds)\ge x, N(s,s+ds]=0\right) . \end{aligned}$$
  Note that $T_{N_{s+ds}^{x}}^{x}\in (s, s+ds]$ and $\frac{dU(t)}{dt}=g(U(t))$, for any $t\in (s,s+ds]$. By Lemma 1, we have
  $$\begin{aligned} |U(s+ds)-U(s)-g(x)ds)|\le KK_{1}d^{2}s. \end{aligned}$$
  Hence, when $s>0$, we get
  $$\begin{aligned}&P^{u}(U(s)<x, U(s+ds)\ge x, N(s,s+ds]=0)\nonumber \\&\quad =P^{u}(x-g(x)ds+O(d^{2}s)\le U(s)<x, N(s,s+ds]=0)\nonumber \\&\quad =P^{u}(x-g(x)ds+O(d^{2}s)\le U(s)<x)e^{-\lambda ds}\nonumber \\&\quad = g(x)p(s,u,x)ds+O(d^{2}s), \end{aligned}$$
  and
  $$\begin{aligned} \sum \limits _{k=1}^{\infty }P^{u}\left( T_{k}^{x}\in ds, N(s,s+ds]\ge 1\right) =O(d^{2}s). \end{aligned}$$
  Then we have
  4 $$\begin{aligned} g_{u}^{x}(s)=g(x)p(s,u,x). \end{aligned}$$
  When $s=0$, we get
  $$\begin{aligned} g_{u}^{x}(0)ds&= {} \sum \limits _{k=1}^{\infty }P^{u}\left( T_{k}^{x}\in (0,ds]\right) =\sum \limits _{k=1}^{\infty }P^{u}\left( T_{k}^{x}\in ds\right) \\&= {} \sum \limits _{k=1}^{\infty }P^{u}\left( T_{k}^{x}\in ds, N(0,ds]=0\right) +\sum \limits _{k=1}^{\infty }P^{u}\left( T_{k}^{x}\in ds, N(0,ds]\ge 1\right) \\&= {} O(d^{2}s). \end{aligned}$$
  Hence we arrive at
  5 $$\begin{aligned} g_{u}^{x}(0)=0. \end{aligned}$$
  Combining (4) with (5), we obtain (3) immediately.
2. when $\phi (s,u)<x$, $P^{u}(T_{1}^{x}>s)=1$. This follows that $g_{u}^{x}(s)=0$.
3. when $\phi (s,u)=x$. (a) If $0\le t<s$, then $\phi (t,u)<x$, thus $G_{u}^{x}(t)=0$. If $t=s$, then there is no jump before *s*, that is
  $$\begin{aligned} G_{u}^{x}(s)=P^{u}(T_{1}^{x}=s)=P^{u}(S_{1}>s)=e^{-\lambda s}. \end{aligned}$$
  (b) When $s=0, u=x$, we have
  $$\begin{aligned} g_{u}^{x}(0)ds&= {} \sum \limits _{k=1}^{\infty }P^{u}(T_{k}^{x}\in (0,ds])=\sum \limits _{k=1}^{\infty }P^{u}(T_{k}^{x}\in (0, ds], N(0,ds]\ge 1)=O(d^{2}s). \end{aligned}$$
  Hence, $g_{u}^{x}(0)=0$. So the proof is completed. $\square$

### *Remark 1*

When $g(x)=c$, the risk model is reduced to the classical risk model, Theorem 1 coincides exactly with Lemma 3.1 in Wu et al. (2003).

For the development of the paper, let us present some Laplace–Stieltjes ($L-S$) transforms, which can be expressed as:

$$\begin{aligned} {\widehat{G}}_{u}^{x}(v)=\int _{0}^{\infty }e^{-vs}dG_{u}^{x}(s),\\ {\widehat{G}}_{x}^{x}(v)=\int _{0}^{\infty }e^{-vs}dG_{x}^{x}(s),\\ {\widehat{F}}_{u}^{x}(v)=\int _{0}^{\infty }e^{-vs}dF_{x}^{x}(s). \end{aligned}$$

### **Lemma 2**

*There exists a constant* *M*, *such that*

$$\begin{aligned} {\widehat{G}}_{x}^{x}(v)=\int _{0}^{\infty }e^{-vs}dG_{x}^{x}(s)<1, \end{aligned}$$

*for any* $$v\ge M$$.

### *Proof*

By Theorem 1 and its proof, we have $G_{x}^{x}(0)=0$ and $T_{n}^{x}\ge S_{n}$. Hence we have

$$\begin{aligned} G_{x}^{x}(t)=\sum \limits _{k=1}^{\infty }P^x(T_{k}^{x}\le t)\le \sum \limits _{k=1}^{\infty }P^x(S_{k}\le t)=\lambda t. \end{aligned}$$

Note that

$$\begin{aligned} {\widehat{G}}_{x}^{x}(v)&= {} \int _{0}^{\infty }e^{-vs}dG_{x}^{x}(s)\\&= {} G_{x}^{x}(s)e^{-vs}|_{0}^{\infty } +v\int _{0}^{\infty }e^{-vs}G_{x}^{x}(s)ds\\&\le {} v\int _{0}^{\infty }e^{-vs}\lambda s ds=\frac{\lambda }{v}. \end{aligned}$$

When $M>\lambda$, we have

$$\begin{aligned} {\widehat{G}}_{x}^{x}(v)=\int _{0}^{\infty }e^{-vs}dG_{x}^{x}(s)\le \frac{\lambda }{v}<1,\quad \mathrm{{for\ any }}\ v>M. \end{aligned}$$

So we complete the proof. $\square$

By Theorem 1, we see that the expression of the renewal measure $G_{u}^{x}$ can be derived once *u* and *x* are fixed. Next, we consider the explicit expressions of the distribution on the first hitting time, which is expressed in terms of $G_{u}^{x}$.

### **Theorem 2**

*For* $s>0$, *we have*

$$\begin{aligned} F_{u}^{x}(s)=\sum \limits _{n=0}^{\infty }(-1)^{n}\left( G_{x}^{x}\right) ^{ n*}*G_{u}^{x}(s). \end{aligned}$$

### *Proof*

By (2) we have the following defective renewal equation

6 $$\begin{aligned} G_{u}^{x}(t)=F_{u}^{x}(t)+F_{u}^{x}*G_{x}^{x}(t). \end{aligned}$$

Taking $L-S$ transform on both sides of (6), we get

$$\begin{aligned} {\widehat{F}}_{u}^{x}(v)=\frac{{\widehat{G}}_{u}^{x}(v)}{1+{\widehat{G}}_{x}^{x}(v)}. \end{aligned}$$

From this together with Lemma 2, we obtain

$$\begin{aligned} {\widehat{F}}_{u}^{x}(v)=\sum \limits _{n=0}^{\infty }(-1)^{n}{\widehat{G}}_{u}^{x}(v)\left[ {\widehat{G}}_{x}^{x}(v)\right] ^{n}. \end{aligned}$$

Inverting ${\widehat{F}}_{u}^{x}(v)$, we have

$$\begin{aligned} F_{u}^{x}(s)=\sum \limits _{n=0}^{\infty }(-1)^{n}\left( G_{x}^{x}\right) ^{ n*}*G_{u}^{x}(s). \end{aligned}$$

This completes the proof. $\square$

### **Corollary 1**

7 $$\begin{aligned}&\Psi (u)=1-\Phi (u)=\sum \limits _{n=0}^{\infty }(-1)^{n}\left( G_{0}^{0}\right) ^{ n*}*G_{u}^{0}(\infty ),\end{aligned}$$

8 $$\begin{aligned}&\Psi _{x}(u)=1-\Phi _{x}(u)=\sum \limits _{n=0}^{\infty }(-1)^{n}\left( G_{x}^{x}\right) ^{ n*}*G_{u}^{x}(\infty ). \end{aligned}$$

### *Proof*

Since $P^{u}(\lim \nolimits _{t\rightarrow \infty }U(t)=\infty )=1$, then $\Psi (u)=P^{u}(T<\infty )=P^{u}(T_{1}^{0}<\infty )$ and $\Psi _{x}(u)=P^{u}(T_{x}<\infty )=P^{u}(T_{1}^{x}<\infty )$ can be obtained directly. $\square$

## The supreme profit and the deficit

In this section, some distributions on the maximum surplus and the maximal severity of ruin are given. Before proceeding with the next Theorem, we will give a simple explanation of shift operators first. For $t\ge 0$, let $\theta _{t}$ be the shift operators from $\Omega$ to itself defined by $U(s)\circ \theta _{t}= U(s+t)$. For stopping time *T*, conditioning on $\{T<\infty \}$ the map $\theta _{T}$ is defined by $U(t)\circ \theta _{T}=U(t+T)$ (see Revuz and Yor 1991, pp. 34, 37 and 74). Let $G(u,a)=P^{u}(\sup \nolimits _{0\le t<T}U(t)>a,T<\infty )$ to denote the probability distribution of the supreme profit of an insurance company before the time of ruin. First we will give the explicit expression of *G*(*u*, *a*).

### **Theorem 3**

*For* $u\ge 0$, *we have*

$$\begin{aligned} G(u,a) =\left\{ \begin{array}{ll} \Psi (u),&{}\quad a\le u,\\ \frac{\Phi (u)}{\Phi (a)}\Psi (a),&{}\quad a>u,\\ \end{array}\right. \end{aligned}$$

*where* $\Psi (u), \Phi (u)$ *are given by* (7).

### *Proof*

When $a\le u$, it is obvious that $G(u,a)=P^{u}(T<\infty )=\Psi (u)$. Therefore we only consider the case $a>u$. Since $P^{u}(\lim \nolimits _{t\rightarrow \infty }U(t)=\infty )=1$, by the strong Markov property of $\{U(t),t\ge 0\}$ we can show that

9 $$\begin{aligned} G\left( u,a\right)&= {} P^{u}\left( T_{1}^{a}<T, T<\infty \right) =P^{u}\left( T_{1}^{a}<T,T\circ \theta _{T_{1}^{a}}<\infty \right) \nonumber \\&= {} P^{u}\left( T_{1}^{a}<T\right) P^{a}\left( T<\infty \right) =P^{u}\left( T_{1}^{a}<T\right) \Psi (a). \end{aligned}$$

In addition, we have

10 $$\begin{aligned} P^{u}\left( T_{1}^{a}<T\right)&= {} P^{u}\left( T_{1}^{a}<T, T<\infty \right) +P^{u}\left( T_{1}^{a}<T, T=\infty \right) \nonumber \\&= {} G(u,a)+\Phi (u). \end{aligned}$$

Inserting (10) into (9), we get that

11 $$\begin{aligned} G(u,a)=\frac{\Psi (a)}{1-\Psi (a)}\Phi (u)=\frac{\Phi (u)}{\Phi (a)}\Psi (a). \end{aligned}$$

This ends the proof. $\square$

### **Corollary 2**

*For* $a>u\ge 0$, *we have*

$$\begin{aligned} P^{u}(T_{1}^{a}<T)=\frac{\Phi (u)}{\Phi (a)}. \end{aligned}$$

### *Proof*

This follows from (10) and (11). $\square$

### **Theorem 4**

*For* $a>u$, *we have*

$$\begin{aligned} P^{u}\Big (\sup \limits _{0\le t<L}U(t)<a,L>0\Big )=\left\{ \begin{array}{ll} \Phi (a)-\Phi (u),&{}\quad u\ge 0,\\ \Phi (a),&{}\quad u<0,\\ \end{array}\right. \end{aligned}$$

*where* $\Phi (u)$ *can be obtained by* (7).

### *Proof*

It follows from $P^{u}(\lim \nolimits _{t\rightarrow \infty }U(t)=\infty )=1$ that $P^{u}(L<\infty )=1$ and $P^{u}(T_{1}^{a}<\infty )=1$. Note that

$$\begin{aligned} \left( \sup \limits _{0\le t<L}U(t)<a,L>0\right) =\left( L<T_{1}^{a},L>0\right) =\left( T\circ \theta _{T_{1}^{a}}=\infty ,L>0\right) . \end{aligned}$$

When $u<0$, we have $P^{u}(L>0)=1$. Thus we can obtain

$$\begin{aligned} P^{u}\left( L<T_{1}^{a},L>0\right)&= {} P^{u}\left( T\circ \theta _{T_{1}^{a}}=\infty \right) =P^{u}\left( T_{1}^{a}<\infty ,T\circ \theta _{T_{1}^{a}}=\infty \right) \\&= {} P^{a}(T=\infty )=\Phi (a). \end{aligned}$$

When $u\ge 0$, we have $(L>0)=(T<\infty )$, then it is easy to see that

$$\begin{aligned}&P^{u}\left( L<T_{1}^{a},L>0\right) =P^{u}\left( T_{1}^{a}<\infty ,T<\infty ,T\circ \theta _{T_{1}^{a}}=\infty \right) \\&\quad =P^{u}\left( T_{1}^{a}<\infty ,T<T_{1}^{a},T\circ \theta _{T_{1}^{a}}=\infty \right) =P^{u}\left( T<T_{1}^{a}\right) \Phi (a). \end{aligned}$$

By Corollary 2, we have $P^{u}(T<T_{1}^{a})=\frac{\Phi (a)-\Phi (u)}{\Phi (a)}$. Hence $P^{u}(L<T_{1}^{a},L>0)=\Phi (a)-\Phi (u)$. This completes the proof. $\square$

In the following, we consider the maximum surplus and the maximal severity of ruin before the time of recovery. To some extent, as ‘indexes’, they can describe the ‘best’ situation and the ‘worst’ situation the company would experience before the surplus process up-crossing level zero after ruin for the first time. Their joint distributions are derived.

### **Theorem 5**

*For* $a>u\ge 0$ *and* $b>0$, *we have*

$$\begin{aligned} P^{u}\Big (\sup \limits _{0\le t<T_{1}^{0}}U(t)<a,\inf \limits _{0\le t<T_{1}^{0}}U(t)\ge -b\Big )=\frac{\Phi (a)\Phi _{-b}(u)-\Phi (u)\Phi _{-b}(a)}{\Phi (a)\Phi _{-b}(0)}, \end{aligned}$$

*where* $\Phi (u), \Phi _{-b}(u)$ *are presented by* (7) *and* (8).

### *Proof*

Let $A= \{\sup \nolimits _{0\le t<T_{1}^{0}}U(t)\ge a,\inf \nolimits _{0\le t<T_{1}^{0}}U(t)\ge -b\}$, and then we have

$$\begin{aligned} P^{u}(A\cap \{T=\infty \})&= {} P^{u}\Big (\sup \limits _{t\ge 0}U(t)\ge a,\inf \limits _{t\ge 0}U(t)\ge -b,\inf \limits _{t\ge 0}U(t)\ge 0\Big )\\&= {} P^{u}\Big (\inf \limits _{t\ge 0}U(t)\ge 0\Big )=\Phi (u), \end{aligned}$$

$$\begin{aligned} P^{u}(A\cap \{T<\infty \})&= {} P^{u}\Big (\sup \limits _{0\le t<T}U(t)\ge a,\inf \limits _{T\le t<T_{1}^{0}}U(t)\ge -b,T<\infty \Big )\\&= {} P^{u}\Big (T_{1}^{a}<T,\Big (\inf \limits _{T\le t<T_{1}^{0}}U(t)\ge -b,T<\infty \Big )\circ \theta _{T_{1}^{a}}\Big )\\&= {} P^{u}\left( T_{1}^{a}<T\right) P^{a}\Big (\inf \limits _{T\le t<T_{1}^{0}}U(t)\ge -b,T<\infty \Big ). \end{aligned}$$

Following the line of Picard (1994), we introduce $\widetilde{M}=Max\{|U(t)|,U(t)<0\}$ in order to obtain $M=Max\{|U(t)|,T\le t<T_{1}^{0}\}$. Clearly $\widetilde{M}\le z$ means that the surplus process never goes under the level $-z$, so that

$$\begin{aligned} P^{u}(\widetilde{M}\le z)=1-\Psi _{-z}(u),\quad z\ge 0. \end{aligned}$$

The event $\widetilde{M}\le z$ is equivalent to that ruin does not occur or ruin occurs and $\widetilde{M}\le z$. By the total probability formula, we have

$$\begin{aligned} P^{u}(\widetilde{M}\le z)=1-\Psi (u)+P^{u}(M\le z)(1-\Psi _{-z}(0)). \end{aligned}$$

Hence we obtain

$$\begin{aligned} P^{u}(M\le z)=\frac{\Phi _{-z}(u)-\Phi (u)}{\Phi _{-z}(0)}. \end{aligned}$$

Then we get

$$\begin{aligned} P^{a}\Big (\inf \limits _{T\le t<T_{1}^{0}}U(t)\ge -b,T<\infty \Big )= P^{a}(M\le b)=\frac{\Phi _{-b}(a)-\Phi (a)}{\Phi _{-b}(0)}. \end{aligned}$$

By Corollary 2, we have

$$\begin{aligned} P^{u}\Big (\sup \limits _{0\le t<T_{1}^{0}}U(t)\ge a,\inf \limits _{0\le t<T_{1}^{0}}U(t)\ge -b\Big )=P^{u}(A)=\Phi (u)+\frac{\Phi (u)(\Phi _{-b}(a)-\Phi (a))}{\Phi (a)\Phi _{-b}(0)}. \end{aligned}$$

Using similar argument, we obtain

$$\begin{aligned}&P^{u}\Big (\inf \limits _{0\le t<T_{1}^{0}}U(t)\ge -b,T=\infty \Big )=\Phi (u),\\&P^{u}\Big (\inf \limits _{0\le t<T_{1}^{0}}U(t)\ge -b,T<\infty \Big )=P^{u}\Big (\inf \limits _{T\le t<T_{1}^{0}}U(t)\ge -b,T<\infty \Big ) =\frac{\Phi _{-b}(u)-\Phi (u)}{\Phi _{-b}(0)}. \end{aligned}$$

Therefore, we can get

$$\begin{aligned}&P^{u}\Big (\sup \limits _{0\le t<T_{1}^{0}}U(t)<a,\inf \limits _{0\le t<T_{1}^{0}}U(t)\ge -b\Big )\\&\quad =P^{u}\Big (\inf \limits _{0\le t<T_{1}^{0}}U(t)\ge -b\Big )-P^{u}\Big (\sup \limits _{0\le t<T_{1}^{0}}U(t)\ge a,\inf \limits _{0\le t<T_{1}^{0}}U(t)\ge -b\Big )\\&\quad =\frac{\Phi (a)\Phi _{-b}(u)-\Phi (u)\Phi _{-b}(a)}{\Phi (a)\Phi _{-b}(0)}. \end{aligned}$$

This ends the proof. $\square$

### *Remark 2*

1. When $g(x)=c$, the risk model simplifies to the classical compound Poisson risk model, Theorem 5 is the same as Lemma 3.5 in Wu et al. (2003).
2. When $g(x)=c, a=\infty$, Theorem 5 simplifies to
  $$\begin{aligned} P^{u}\Big (\inf \limits _{0\le t<T_{1}^{0}}U(t)\ge -b\Big )=\frac{\Phi (u+b)-\Phi (u)}{\Phi (b)}, \end{aligned}$$
  which coincides with Theorem 1 in Picard (1994).
3. When $g(x)=c+\delta x$ for $x \ge 0$ and $g(x)=c+r x$ for $x<0$, the risk model is reduced to the risk model with credit and debit interests. Let $a=\infty$, Theorem 5 simplifies to
  $$\begin{aligned} P^{u}\Big (\inf \limits _{0\le t<T_{1}^{0}}U(t)\ge -b\Big )=\frac{\Phi _{-b}(u)-\Phi (u)}{\Phi _{-b}(0)}, \end{aligned}$$
  which is the same as (6.2) in Li and Lu (2013).

Next, we consider the maximum surplus and the maximal severity of ruin before the time of the surplus process leaving zero ultimately, which describe the best situation and the worst situation the company would experience before the time of the surplus process leaving zero ultimately. We obtain their explicit expression in the following theorem.

### **Theorem 6**

*For* $a>u\ge 0$ *and* $b>0$, *we have*

$$\begin{aligned} P^{u}\Big (\sup \limits _{0\le t<L}U(t)<a,\inf \limits _{0\le t<L}U(t)\ge -b,L>0\Big )=\frac{\Phi _{-b}(u)}{\Phi _{-b}(a)}\Phi (a)-\Phi (u), \end{aligned}$$

*In particular*,

$$\begin{aligned}&P^{u}\Big (\inf \limits _{0\le t<L}U(t)\ge -b,L>0\Big )=\Phi _{-b}(u)-\Phi (u),\\&P^{u}\Big (\sup \limits _{0\le t<L}U(t)<a,L>0\Big )=\Phi (a)-\Phi (u), \end{aligned}$$

*where* $\Phi (u), \Phi _{-b}(u)$ *are given by* (7) and (8).

### *Proof*

Note that the event $\{\sup \nolimits _{0\le t<L}U(t)<a,\inf \nolimits _{0\le t<L}U(t)\ge -b,L>0\}$ is equivalent to the event $\{T<T_{1}^{a}<T_{-b},\inf \nolimits _{t\ge 0}U(T_{1}^{a}+t)\ge 0\}$, so we can obtain

$$\begin{aligned}&P^{u}\Big (\sup \limits _{0\le t<L}U(t)<a,\inf \limits _{0\le t<L}U(t)\ge -b,L>0\Big )\\&\quad =P^{u}\Big (T<T_{1}^{a}<T_{-b},\inf \limits _{t\ge 0}U(T_{1}^{a}+t)\ge 0\Big )\\&\quad = P^{u}\Big (T_{1}^{a}<T_{-b},\inf \limits _{t\ge 0}U(T_{1}^{a}+t)\ge 0\Big )-P^{u}\Big (T_{1}^{a}<T,\inf \limits _{t\ge 0}U(T_{1}^{a}+t)\ge 0\Big )\\&\quad =P^{u}\Big [P^{u}\Big (T_{1}^{a}<T_{-b},\inf \limits _{t\ge 0}U(T_{1}^{a}+t)\ge 0|{\mathscr {F}}_{T_{1}^{a}}\Big )\Big ]\\&\qquad -P^{u}\Big [P^{u}\Big (T_{1}^{a}<T,\inf \limits _{t\ge 0}U(T_{1}^{a}+t)\ge 0|{\mathscr {F}}_{T_{1}^{a}}\Big )\Big ]\\&\quad =P^{u}(T_{1}^{a}<T_{-b})P^{a}\Big (\inf \limits _{t\ge 0}U(t)\ge 0\Big )-P^{u}\left( T_{1}^{a}<T\right) P^{a}\Big (\inf \limits _{t\ge 0}U(t)\ge 0\Big ). \end{aligned}$$

Using the same argument as Theorem 3 and Corollary 2, we have

$$\begin{aligned} P^{u}(T_{1}^{a}<T_{-b})=\frac{\Phi _{-b}(u)}{\Phi _{-b}(a)},\quad P^{u}(T_{1}^{a}<T)=\frac{\Phi (u)}{\Phi (a)}. \end{aligned}$$

Hence we can obtain

$$\begin{aligned} P^{u}\left( \sup \limits _{0\le t<L}U(t)<a,\inf \limits _{0\le t<L}U(t)\ge -b,L>0\right) =\frac{\Phi _{-b}(u)}{\Phi _{-b}(a)}\Phi (a)-\Phi (u). \end{aligned}$$

This completes the proof. $\square$

### *Example 1*

Let $g(x)=c$ and the individual claim amount distribution be exponential with mean value $\mu$. Then

$$\begin{aligned} f^{n*}(x)=\frac{1}{\mu ^{n}\Gamma (n)}x^{n-1}e^{-\frac{x}{\mu }}, \quad x>0, \end{aligned}$$

and

$$\begin{aligned} p(s,u,x)&= {} e^{-\lambda s}\sum \limits _{n=1}^{\infty }\frac{(\lambda s)^{n}}{n!}f^{n*}(u+cs-x)\\&= {} \frac{\lambda s}{\mu }e^{-\frac{u-x+(c+\lambda \mu )s}{\mu }} \quad \sum \limits _{n=1}^{\infty }\left[ \frac{1}{n \Gamma ^{2}(n)}\left( \frac{\lambda s(u-x+cs)}{\mu }\right) ^{n-1}\right] . \end{aligned}$$

Thus

$$\begin{aligned} g_{u}^{x}(s)=\frac{\lambda cs}{\mu } e^{-\frac{u-x+(c+\lambda \mu )s}{\mu }}\quad\sum \limits _{n=1}^{\infty }\left[ \frac{1}{n \Gamma ^{2}(n)}\left( \frac{\lambda s(u-x+cs)}{\mu }\right) ^{n-1}\right] . \end{aligned}$$

Hence

$$\begin{aligned} f_{u}^{x}(s)=&\frac{1}{s}e^{-\frac{u-x+(c+\lambda \mu )s}{\mu }}\quad\sum \limits _{n=0}^{\infty }(-1)^{n}\sum \limits _{m_{1}=1}^{\infty }\cdots \sum \limits _{m_{n}=1}^{\infty }\sum \limits _{m=1}^{\infty } \sum \limits _{k=1}^{m-1}\left( \frac{\lambda cs^{2}}{\mu }\right) ^{\sum \limits _{i=1}^{n}m_{i}}\\&\quad \times \prod \limits _{i=1}^{n}\frac{B(2\sum \nolimits _{k=1}^{i-1}m_{k},2m_{i})}{m_{i} \Gamma ^{2}(m_{i})}\left( \frac{\lambda s}{\mu }\right) ^{m} \frac{B(2\sum \nolimits _{i=1}^{n}m_{i},m+k-1)}{m \Gamma ^{2}(m)}C_{m-1}^{k}(u-x)^{m-1-k}(cs)^{k+1}, \end{aligned}$$

where $B(x,y)=\int _{0}^{1}t^{x-1}(1-t)^{y-1}dt,x>0,y>0$ is the Beta-function and $C_{n}^{m}=\frac{n!}{m!(n-m)!}$ is a combinatorial number. It follows that

$$\begin{aligned} \Psi _{x}(u)=\frac{\lambda \mu }{c}e^{-\frac{(c-\lambda \mu)(u-x)}{c\mu}},\quad \Psi (u)=\frac{\lambda \mu }{c}e^{-\frac{(c-\lambda \mu )u}{c\mu }}, \end{aligned}$$

and

$$\begin{aligned} \Phi _{x}(u)&= {} 1-\Psi _{x}(u)=1-\frac{\lambda \mu }{c}e^{-\frac{(c-\lambda \mu )(u-x)}{c\mu }},\\ \Phi (u)&= {} 1-\Psi (u)=1-\frac{\lambda \mu }{c}e^{-\frac{(c-\lambda \mu )u}{c\mu }}. \end{aligned}$$

By Theorem 3, we have

$$\begin{aligned} G(u,a) =\left\{ \begin{array}{ll} \frac{\lambda \mu }{c}e^{-\frac{(c-\lambda \mu )u}{c\mu }},&{}\quad a\le u,\\ \frac{\lambda \mu e^{-\frac{(c-\lambda \mu )a}{c\mu }}\quad\left( c-\lambda \mu e^{-\frac{(c-\lambda \mu )u}{c\mu }}\right ) }{c \left( c-\lambda \mu e^{-\frac{(c-\lambda \mu )a}{c\mu }}\right) }, &{}\quad a>u.\\ \end{array}\right. \end{aligned}$$

By Theorem 4, we get

$$\begin{aligned} P^{u}\Big (\sup \limits _{0\le t<L}U(t)<a,L>0\Big )=\left\{ \begin{array}{ll} \frac{\lambda \mu }{c}\left( e^{-\frac{(c-\lambda \mu )u}{c\mu }}-e^{-\frac{(c-\lambda \mu )a}{c\mu }}\right) ,&{}\quad u\ge 0,\\ 1-\frac{\lambda \mu }{c}e^{-\frac{(c-\lambda \mu )a}{c\mu }},&{}\quad u<0.\\ \end{array}\right. \end{aligned}$$

By Theorem 5, we have

$$\begin{aligned}&P^{u}\Big (\sup \limits _{0\le t<T_{1}^{0}}U(t)<a,\inf \limits _{0\le t<T_{1}^{0}}U(t)\ge -b\Big )\\&\quad =\frac{\left( c-\lambda \mu e^{-\frac{(c-\lambda \mu )(u+b)}{c\mu }}\right) }{\left( c-\lambda \mu e^{-\frac{(c-\lambda \mu )b}{c\mu }}\right) }- \frac{\left( c-\lambda \mu e^{-\frac{(c-\lambda \mu )u}{c\mu }}\right) \left( c-\lambda \mu e^{-\frac{(c-\lambda \mu )(a+b)}{c\mu }}\right) }{ \left( c-\lambda \mu e^{-\frac{(c-\lambda \mu )a}{c\mu }}\right) \left( c-\lambda \mu e^{-\frac{(c-\lambda \mu )b}{c\mu } }\right) }. \end{aligned}$$

When $u=0$, the result is the same as *W*(0, *a*, *b*) in Wu et al. (2003). By Theorem 6, we have

$$\begin{aligned}&P^{u}\Big (\sup \limits _{0\le t<L}U(t)<a,\inf \limits _{0\le t<L}U(t)\ge -b,L>0\Big )\\&\quad =\frac{\left( c-\lambda \mu e^{-\frac{(c-\lambda \mu )(u+b)}{c\mu } }\right) }{\left( c-\lambda \mu e^{-\frac{(c-\lambda \mu )(a+b)}{c\mu }}\right) }\left( 1-\frac{\lambda \mu }{c}e^{-\frac{(c-\lambda \mu )a}{c\mu }}\right) -\left( 1-\frac{\lambda \mu }{c}e^{-\frac{(c-\lambda \mu )u}{c\mu }}\right) . \end{aligned}$$

## Conclusions

In order to make a reasonably realistic description of the actual behavior, we investigate the risk model whose income depend on the current reserve.

The distribution of extremum is very important in risk theory, which can portray the best and worst condition of an insurance company. The research of extremum is necessary and meaningful whether theoretically or practically. In this paper, the distribution of supreme profits before ruin, the joint distributions of the supreme profit and the deficit before the time of the surplus process first up-crossing level zero after ruin, and the joint distributions of the supreme profit and the deficit before the surplus process leave zero ultimately are derived. All these results provide early warning for the development of the insurance company.

Concretely speaking, the distributions of some extremum can be converted to the problem of hitting time, so we study the first hitting time of this model. With the help of the strong Markov property, we construct the renewal measure of the defective renewal sequence, and obtain the distribution of the renewal measure. The method that we used to solve the stopping time problem is innovative. By the presented renewal measure and the Laplace–Stieltjes transforms, the distribution of the first hitting time is obtained explicitly. Then, the ruin probability and the probability that the surplus process less than *x* is obtained.
